# Association between Psychiatric disorders & Substance use disorder in rehabilitation center of Islamabad: A cross-sectional study

**DOI:** 10.1192/j.eurpsy.2022.2126

**Published:** 2022-09-01

**Authors:** A. Berkat, M. Abidi

**Affiliations:** 1 Fresh Start Rehab and caring center, Psychiatry, Islamabad, Pakistan; 2 The Brain clinic, Psychiatry, Karachi, Pakistan

**Keywords:** substance, delusion, Mental, Hallucination

## Abstract

**Introduction:**

The prevalence of substance use disorder has rapidly increased recently. It is believed that the occurrence of mental disorders is strongly associated with substance use.

**Objectives:**

To identify prevelance of different psychiatric mobidity & symptomatology as Comorbidity amon the diagnosed patients of Substance Use Disorder

**Methods:**

This study was conducted from June till December 2021. A total of 486 PDUs were recruited for this study. A self-administered questionnaire was distributed among PDUs admitted at the Rehabilitation Centre during the period of the study. The questionnaire inquired about the demographic details of the PDUs, their substance history and the occurrence of any MDs.

**Results:**

The mean age of the PDUs was 25.9 + 6.0 years. A total of (95%) men and (5%) women reported their gender. There were single (74.7%), married (23.1%), divorced (1.4%) and separated (0.7%) PDUs. A large majority of the PDUs (n = 159, 55.6%) had been using different drugs for more than three years. The various MDs reported among the PDUs were delusion (n = 100, 35.2%); paranoia (n = 51, 17.8%); mania (n = 36, 12.6%); depression (n = 156, 54.5%); (n = 100, 35.2%); auditory hallucinations (n = 73, 25.7%); visual hallucinations (n = 106, 37.3%) and anxiety (n = 46, 16.2%). Among 164 cannabis users, hallucinations (n = 35, 21.3%; p = 0.04) was the only significant MD.

**Conclusions:**

Delusion and paranoia were amongst the most highly prevalent MDs reported. The occurrence of auditory hallucinations, mania and paranoia were significantly associated with cannabis, heroin and cocaine use, respectively.

**Disclosure:**

No significant relationships.

